# Evaluation of ion/electron beam induced deposition for electrical connection using a modern focused ion beam system

**DOI:** 10.1186/s42649-019-0008-2

**Published:** 2019-07-18

**Authors:** Byeong-Seon An, Yena Kwon, Jin-Su Oh, Yeon-Ju Shin, Jae-seon Ju, Cheol-Woong Yang

**Affiliations:** 1 School of Advanced Material Science and Engineering, Sungkyunkwan University, Suwon, 16419 Korea; 2 Cooperative Center for Research Facilities, Sungkyunkwan University, Suwon, 16419 Korea

**Keywords:** Focused ion beam, Electron beam induced deposition, Ion beam induced deposition, Electrical resistance

## Abstract

Focused ion beam method, which has excellent capabilities such as local deposition and selective etching, is widely used for micro-electromechanical system (MEMS)-based in situ transmission electron microscopy (TEM) sample fabrication. Among the MEMS chips in which one can apply various external stimuli, the electrical MEMS chips require connection between the TEM sample and the electrodes in MEMS chip, and a connected deposition material with low electrical resistance is required to apply the electrical signal. Therefore, in this study, we introduce an optimized condition by comparing the electrical resistance for C-, Pt-, and W- ion beam induced deposition (IBID) at 30 kV and electron beam induced deposition (EBID) at 1 and 5 kV. The W-IBID at 30 kV with the lowest electrical resistance of about 30 Ω shows better electrical properties than C- and Pt-IBID electrodes. The W-EBID at 1 kV has lower electrical resistance than that at 5 kV; thus, confirming its potential as an electrode. Therefore, for the materials that are susceptible to ion beam damage, it is recommended to fabricate electrical connections using W-EBID at 1 kV.

## Introduction

Focused ion beam (FIB) has the ability to locally deposit materials, etch a specimen by using the gas injection system, and selectively mill the surface of the specimen through ion sputtering without conventional photomasks (Giannuzzi and Stevie 1999). Therefore, FIB has been widely used to fabricate specimens for transmission electron microscopy (TEM). In particular, FIB sampling method becomes more important in micro-electromechanical system (MEMS)-based in situ TEM (Mele et al. 2016; Vijayan et al. 2017) in which we can observe real-time microstructural changes influenced by external stimuli such as electrical current and thermal and mechanical stress. For in situ TEM with electrical MEMS chips, the formation of a conducting path with low electrical resistance is required to connect TEM specimens to the electrodes in a MEMS chip. The conducting path can be formed either by ion beam induced deposition (IBID) or electron beam induced deposition (EBID) in the FIB (Wilhite et al. 2014). Fawey et al. reported that the electrical resistance of 30 kV IBID is much lower than that of 5 kV EBID for both W and Pt deposition (Hammad Fawey et al. 2016). However, for the samples vulnerable to high energy Ga^+^ ion beam, such as chalcogenide and 2-D materials (An et al. 2018), it is necessary to consider the ion beam damage to samples because it can lead to an unexpected experimental result. Therefore, it is very important to acquire the optimal conditions for IBID and EBID to make a low resistance electrical connection without damaging the TEM specimen. In this study, we introduce the optimized conditions for IBID at 30 kV and EBID at 1 and 5 kV by comparing C, Pt, and W materials.

### Experimental procedure

Experiments were conducted using a Hitachi NX2000 triple-beam FIB system (Hitachi Inc., Japan) equipped with a gas injection system enabling beam-induced depositions with ions or electrons. Metal precursors used for C, Pt, and W materials were phenanthrene (C_14_H_10_), (methylcyclopentadienyl) trimethylplatinum ((CH_3_C_5_H_4_)(CH_3_)_3_Pt), and hexacarbonyltungsten (W (CO)_6_), respectively. The C and W wire electrodes deposited by IBID with nominal dimensions of 16 μm (length) × 5 μm (width) × 0.5 μm (height) were deposited at an accelerating voltage of 30 kV with a current density of 18.7 ρA/μm^2^. Because Pt-IBID with the current density of 18.7 ρA/μm^2^ is etched rather than deposited, the Pt wire electrode was deposited using a current density of 3.5 ρA/μm^2^. The C, Pt, and W electrodes deposited by EBID with nominal dimensions of 13 μm (length) × 2.5 μm (width) × 0.25 μm (height) were fabricated at accelerating voltages of 1 and 5 kV with a current density of 52.3 ρA/um^2^.

After fabricating the electrodes using IBID and EBID, the current-voltage (I-V) characteristics were evaluated using a microprobe system connected to an Agilent B1500A parametric analyzer. I-V curves were measured by applying voltages ranging from − 1.0 to 1.0 V with steps of 25 mV. To analyze the microstructure and chemical composition, cross-sectional TEM specimens were fabricated by FIB using the lift-out technique. The TEM samples were etched using a high-energy Ga^+^ ion beam at 30 keV and 1.5 nA and subsequently thinned between 5 and 10 keV at 40 pA. As the final step, a low-energy Ar^+^ ion beam at 1 keV and 19 nA was used to minimize the damage to the surface layers (Kato 2004). The prepared samples were investigated by analytical TEM (JEM-ARM200F; JEOL, Japan) equipped with energy dispersive X-ray spectroscopy (EDS) and electron energy loss spectroscopy (EELS) operated at 200 kV.

## Results and discussion

**Fig. 1:**
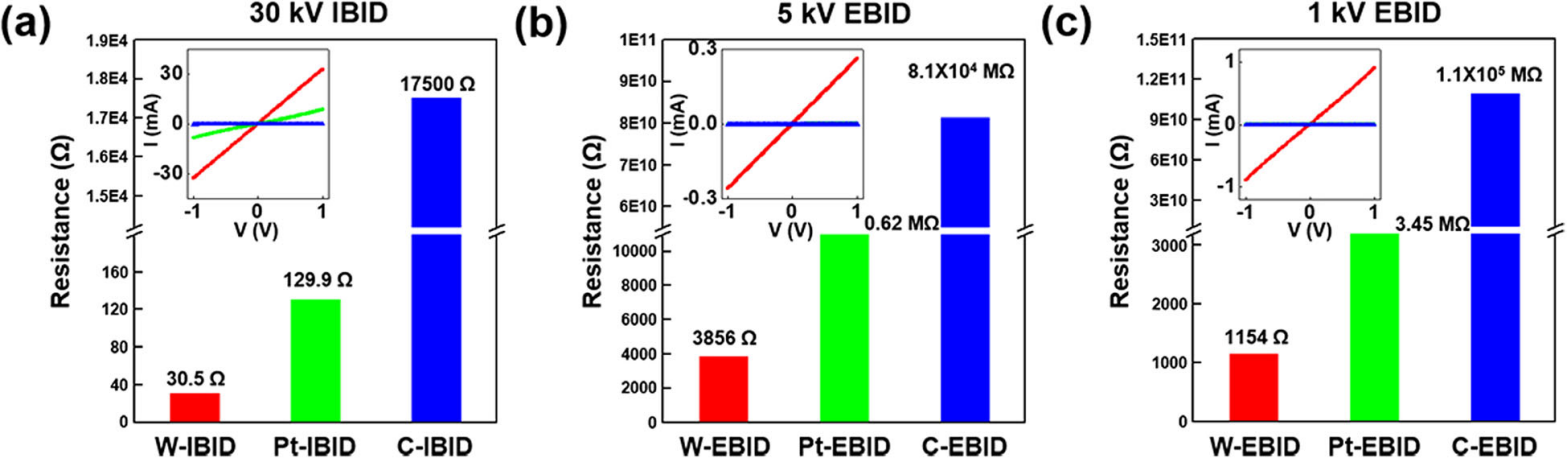
The electrical resistance obtained from I-V curves for (**a**) IBID at 30 kV, (**b**) EBID at 5 kV, and (**c**) EBID at 1 kV

Figure 1 shows the electrical resistance histogram obtained from the I-V curves in the inset for W, Pt, and C wire electrodes deposited by IBID at 30 kV and by EBID at 1 and 5 kV. As shown in Fig. 1a, for IBID at 30 kV, W-IBID exhibited the lowest electrical resistance of about 30 Ω compared to Pt- and C-IBID (129.9 and 17,500 Ω, respectively), and the resistance of C-IBID was approximately three orders of magnitude higher than that of W-IBID. For EBID at 5 kV, the electrical resistance of W-EBID was 3856 Ω, which is one order lower than that of C-IBID at 30 kV. However, the electrical resistances of Pt- and C-EBID at 5 kV were measured to be 0.62 and 8.1 × 10^4^ MΩ, respectively, which are much higher than that of W-EBID as shown in Fig. 1b. On the other hand, for EBID at 1 kV, the W wire electrode was found to have three times lower electrical resistance than W-EBID at 5 kV. The resistances of Pt and C electrodes deposited by EBID at 1 kV were 3.45 and 1.1 × 10^6^ MΩ, respectively (Fig. 1c). Because Pt- and C-EBID at 1 and 5 kV have very high electrical resistances, they are difficult to be used for the electrical connection between the TEM specimen and the electrodes in MEMS chips. W-IBID at 30 kV demonstrated the lowest resistance; however, W-EBID at 1 kV exhibited a lower resistance than EBID at 5 kV irrespective of the depositing material.

**Fig. 2:**
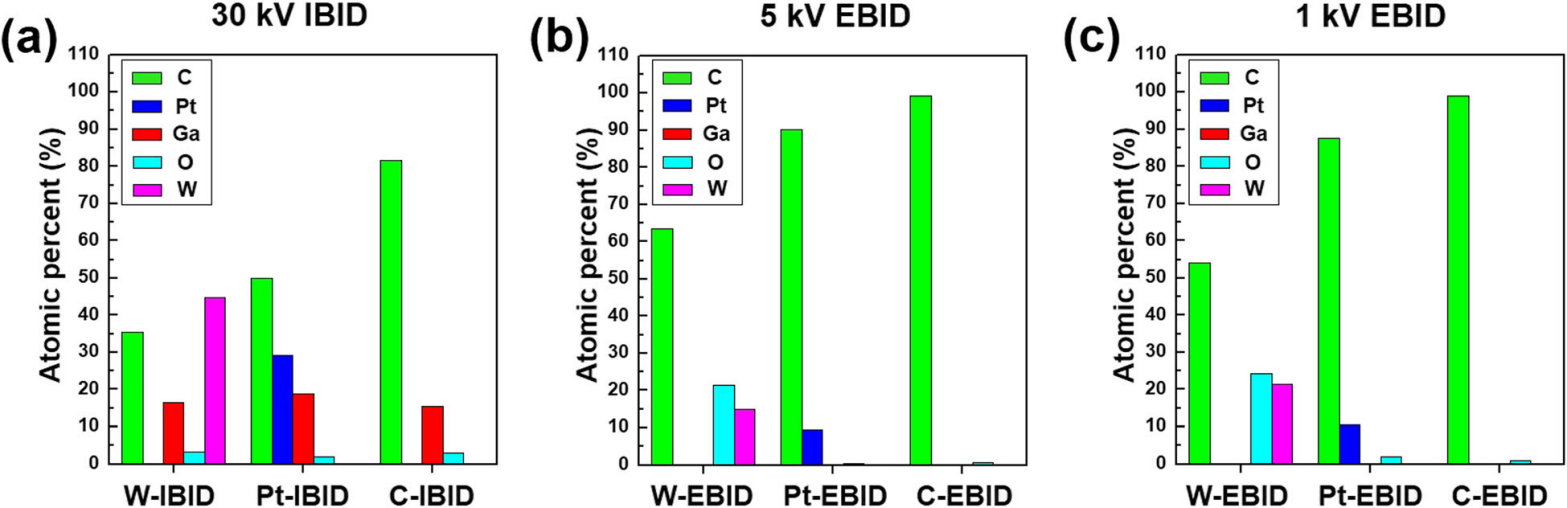
Chemical composition obtained from STEM-EDS analysis for (**a**) IBID at 30 kV, (**b**) EBID at 5 kV, and (**c**) EBID at 1 kV

To assess the difference in electrical resistance of C-, Pt-, and W-IBID and EBID accurately, chemical composition and microstructure analysis using TEM was performed. Figure 2 exhibits the chemical composition obtained from the scanning TEM (STEM)-EDS analyses of the C, Pt, and W wires deposited by IBID and EBID. For IBID, all the wires contain a certain amount of Ga (15.5 to 18.8 at%) because a Ga^+^ ion beam was used for the deposition. For IBID at 30 kV, the W-IBID wire consists of 44.9 at% of W and 35.4 at% of C, while the Pt-IBID wire consists of 29.1 at% of Pt and 50.1 at% of C. Figure 2a shows that W-IBID has a lower C impurity and higher metal content than Pt-IBID. Unlike IBID, for EBID at 1 and 5 kV, no wires contain Ga. The W-EBID at 5 kV was composed of 15 at% of W, 63.5 at% of C, and 21.5 at% of O, whereas that at 1 kV was composed of 21.5 at% of W, 54.1 at% of C, and 24.4 at% of O. The O content in W-EBID can be attributed to the insufficient decomposition of the W(CO)_6_ precursor. The W-EBID at 1 kV has lower impurities and higher metal content than that at 5 kV. However, in the case of Pt- and C-EBID, there was no difference in the composition of wires depending on the accelerating voltage (Fig. 2b and c). Based on the chemical analysis of IBID at 30 kV and EBID at 1 and 5 kV for the different depositing materials, the resistance difference can be attributed to the C content incorporated from the precursor system and some amounts of Ga incorporation.

**Fig. 3:**
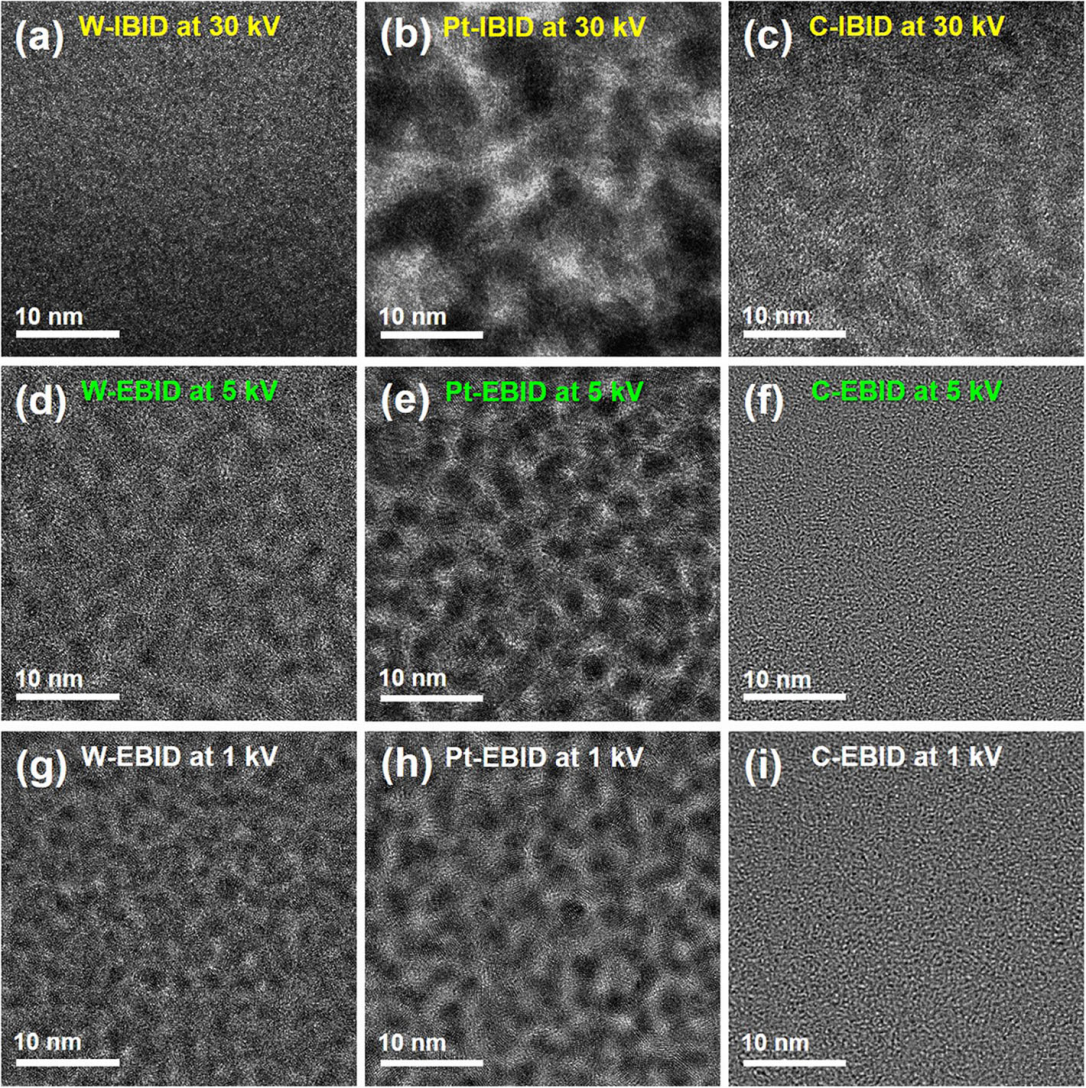
HR-TEM images for (**a**) W-IBID, (**b**) Pt-IBID, and (**c**) C-IBID at 30 kV; (**d**) W-EBID, (**e**) Pt-EBID, and (**f**) C-EBID at 5 kV; and (**g)** W-EBID, (**h**) Pt-EBID, and (**i**) C-EBID at 1 kV

Figure 3a–c show the high-resolution (HR)-TEM images of the C, Pt, and W wires deposited by IBID at 30 kV. The W wire existed in the amorphous form, while the Pt wire was observed in the form of a coarse speckled pattern, and there were crystalline Pt nanoparticles with size of about 10 nm and an amorphous region between the crystalline islands. In addition, the C wire deposited by IBID was also observed to have a speckled pattern due to the distribution of Ga. For the EBID at 1 and 5 kV, there was no noticeable difference in the microstructure with accelerating voltage; however, a distinctive size difference in the microstructure depending on the depositing materials was noticeable. The W-EBID at 1 and 5 kV have very small-sized crystalline W nanoparticles and amorphous impurities agglomerated between the nanoparticles unlike the W-IBID at 30 kV as shown in Fig. 3d and g. Pt nanoparticles in the Pt-EBID at 1 and 5 kV were found to be smaller than those in Pt-IBID (Fig. 3e and h). The impurities present in the amorphous region appear to be C or O. Conversely, C-EBID was observed completely in the amorphous form unlike the C-IBID at 30 kV (Fig. 3f and i).

**Fig. 4:**
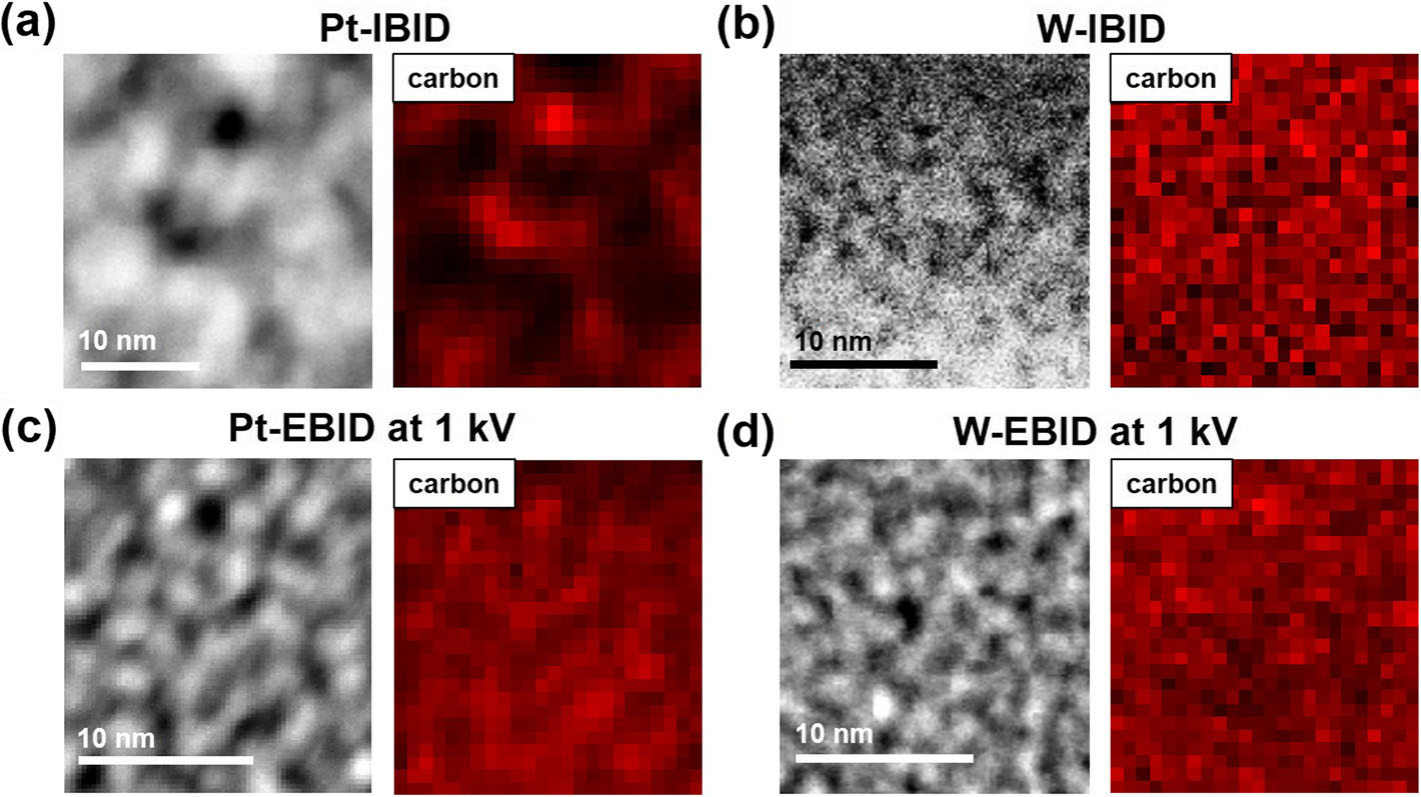
Carbon elemental mapping of STEM-EELS for (**a**) Pt-IBID and (**b**) W-IBID at 30 kV, and (**c**) Pt-EBID and (**d**) W-EBID at 1 kV

To determine accurately whether the impurity in the amorphous region existed in IBID and EBID was C, STEM-EELS was performed to obtain a C elemental map (K edge: 284 eV) for the Pt and W wires deposited by IBID at 30 kV and EBID at 1 kV. As shown in Fig. 4, the crystalline nanoparticle and amorphous impurities appear with strong contrast in the high-angle annular dark-field (HAADF) images obtained via STEM. In the HAADF images, bright and dark regions correspond to the crystalline and amorphous phase, respectively. For Pt-IBID and Pt- and W-EBID, the elemental maps showing the carbon distribution in the bright and dark regions indicate that carbon atoms agglomerate between the crystalline islands as shown in Fig. 4a, c, and d, respectively. On the other hand, as shown in Fig. 4b, W-IBID was observed to have bright and dark regions due to the presence of Ga in the HAADF image. The C impurity in W-EBID was evenly distributed in all regions, which was consistent with the HR-TEM results shown in Fig. 3.

Consequently, from the above results, the W-electrode deposited by IBID and EBID exhibited better electrical performance than the C and Pt electrodes. It is preferable to use W-IBID at 30 kV if the ion beam damage is not considered, while it is recommended to use W-EBID at 1 kV if the sample is susceptible to ion beam damage.

## Conclusions

In this study, we compared the C, Pt, and W electrodes deposited by IBID at 30 kV and EBID at 1 and 5 kV using a Hitachi NX2000 triple beam FIB system. The optimal condition was determined by analyzing the electrical characteristics, microstructure, and chemical composition of the fabricated electrodes using I-V sweep and TEM analyses. As a result, W electrode exhibited a better performance than C and Pt electrodes. It was found that the W- IBID at 30 kV exhibited the lowest resistance of about 30 Ω, and the W-EBID at 1 kV had a lower resistance than the C-, Pt- and W-EBID at 5 kV. Considering the Ga^+^ ion beam damage, it is recommended to create an electrical connection using W-EBID at 1 kV.

## Data Availability

The datasets used and/or analyzed during the current study are available from the corresponding author on reasonable request.

## Abbreviations

EBID: Electron beam induced deposition
EDS: Energy dispersive X-ray spectroscopy
EELS: Electron energy loss spectroscopy
FIB: Focused ion beam
HAADF: High-angle annular dark-field
HR-TEM: High-resolution transmission electron microscopy
IBID: Ion beam induced deposition
MEMS: Micro-electromechanical system
STEM: Scanning transmission electron microscopy
TEM: Transmission electron microscopy
